# Exacerbation of Diabetic Renal Alterations in Mice Lacking Vasohibin-1

**DOI:** 10.1371/journal.pone.0107934

**Published:** 2014-09-25

**Authors:** Norikazu Hinamoto, Yohei Maeshima, Hiroko Yamasaki, Tatsuyo Nasu, Daisuke Saito, Hiroyuki Watatani, Haruyo Ujike, Katsuyuki Tanabe, Kana Masuda, Yuka Arata, Hitoshi Sugiyama, Yasufumi Sato, Hirofumi Makino

**Affiliations:** 1 Department of Medicine and Clinical Science, Okayama University Graduate School of Medicine, Dentistry and Pharmaceutical Sciences, Okayama, Japan; 2 Department of Chronic Kidney Disease and Cardiovascular Disease, Okayama University Graduate School of Medicine, Dentistry and Pharmaceutical Sciences, Okayama, Japan; 3 Department of Chronic Kidney Disease and Peritoneal Dialysis, Okayama University Graduate School of Medicine, Dentistry and Pharmaceutical Sciences, Okayama, Japan; 4 Department of Vascular Biology, Institute of Development, Aging, and Cancer, Tohoku University, Sendai, Japan; National Centre for Scientific Research “Demokritos”, Greece

## Abstract

Vasohibin-1 (VASH1) is a unique endogenous inhibitor of angiogenesis that is induced in endothelial cells by pro-angiogenic factors. We previously reported renoprotective effect of adenoviral delivery of VASH1 in diabetic nephropathy model, and herein investigated the potential protective role of endogenous VASH1 by using VASH1-deficient mice. Streptozotocin-induced type 1 diabetic VASH1 heterozygous knockout mice (VASH1^+/−^) or wild-type diabetic mice were sacrificed 16 weeks after inducing diabetes. In the diabetic VASH1^+/−^ mice, albuminuria were significantly exacerbated compared with the diabetic wild-type littermates, in association with the dysregulated distribution of glomerular slit diaphragm related proteins, nephrin and ZO-1, glomerular basement membrane thickning and reduction of slit diaphragm density. Glomerular monocyte/macrophage infiltration and glomerular nuclear translocation of phosphorylated NF-κB p65 were significantly exacerbated in the diabetic VASH1^+/−^ mice compared with the diabetic wild-type littermates, accompanied by the augmentation of VEGF-A, M1 macrophage-derived MCP-1 and phosphorylation of IκBα, and the decrease of angiopoietin-1/2 ratio and M2 macrophage-derived Arginase-1. The glomerular CD31^+^ endothelial area was also increased in the diabetic VASH1^+/−^ mice compared with the diabetic-wild type littermates. Furthermore, the renal and glomerular hypertrophy, glomerular accumulation of mesangial matrix and type IV collagen and activation of renal TGF-β_1_/Smad3 signaling, a key mediator of renal fibrosis, were exacerbated in the diabetic VASH1^+/−^ mice compared with the diabetic wild-type littermates. In conditionally immortalized mouse podocytes cultured under high glucose condition, transfection of VASH1 small interfering RNA (siRNA) resulted in the reduction of nephrin, angiopoietin-1 and ZO-1, and the augmentation of VEGF-A compared with control siRNA. These results suggest that endogenous VASH1 may regulate the development of diabetic renal alterations, partly via direct effects on podocytes, and thus, a strategy to recover VASH1 might potentially lead to the development of a novel therapeutic approach for diabetic nephropathy.

## Introduction

Diabetic nephropathy is the most common pathological disorder predisposing patients to end-stage renal disease. In the early stage of diabetic nephropathy, glomerular hyperfiltration, glomerular and tubular hypertrophy, microalbuminuria and thickening of the glomerular basement membrane (GBM) are observed. Thereafter, the expansion of the mesangial extracellular matrix and overt proteinuria emerge, thus eventually leading to glomerulosclerosis and tubulointerstitial fibrosis [1]. The involvement of the renin-angiotensin-aldosterone system, chemokines such as monocyte chemoattractant protein-1 (MCP-1)/CCL-2, transforming growth factor-β_1_ (TGF-β_1_) and advanced glycation end products in diabetic nephropathy has been reported [2], [3].

The infiltration of macrophages is associated with diabetic nephropathy [4], [5]. There are at least two types of macrophages, with the M1 macrophages being involved in promoting renal inflammation, and thus being a therapeutic target for renal disease. The other type is M2 macrophages, which are involved in the resolution of inflammation and repair of injury [6].

Angiogenesis is associated with a number of pathological conditions, including tumor growth and diabetic retinopathy [7], and vascular endothelial growth factor-A (VEGF-A) promotes angiogenesis [8] and also induces vascular permeability [9].

Previous studies have demonstrated an increased glomerular filtration surface area in association with the formation of new glomerular capillaries and a slight elongation of the preexisting capillaries in diabetic nephropathy [10], [11], analogous to the findings of pathological diabetic retinopathy [12]. In addition, an increase in the levels of VEGF-A and its receptor (VEGFR-2) has been reported in models of diabetic nephropathy [13], [14]. The therapeutic efficacy of anti-VEGF-A strategies [15], [16] has further demonstrated the potential involvement of VEGF-A in diabetic nephropathy. The therapeutic effects of angiogenesis inhibitors in diabetic nephropathy models have been reported by our group and others [17], [18].

Vasohibin-1 (VASH1) was identified from a microarray analysis that assessed the genes upregulated by VEGF-A in endothelial cells. The human VASH1 protein is composed of 365 amino acid residues and regulates the proliferation and migration of endothelial cells in an autocrine manner and thus is considered to be a negative feedback regulator of angiogenesis [19]. The critical role of VASH1 in the maintenance of endothelial cells against cellular stressors have been reported [20]. However, the cell surface receptor(s) for VASH1 have not yet been identified. The therapeutic efficacy of VASH1 against tumor growth and atherosclerosis models has been reported [19], [21], [22], [23], [24]. We previously reported the therapeutic effects of the adenoviral transfer of human VASH1 in mouse type 1 and 2 diabetic nephropathy models [25], [26]. The renoprotective effects of exogenous VASH1 were mediated via its direct effects on mesangial cells and podocytes, as well as glomerular endothelial cells, thus suggesting that VASH1 has activity beyond its role as an “antiangiogenic factor”.

In the present study, we demonstrate the exacerbation of diabetic nephropathy in VASH1 heterozygous knockout (VASH1^+/−^) mice and reveal the functional role of endogenous VASH1 in the streptozotocin (STZ)-induced type 1 diabetes model. These effects were associated with the regulation of angiogenesis-associated factors, inflammatory signals and podocyte injury, thus potentially leading to the exacerbation of albuminuria.

## Materials and Methods

### Induction of diabetes and experimental protocols

**Figure 1:**
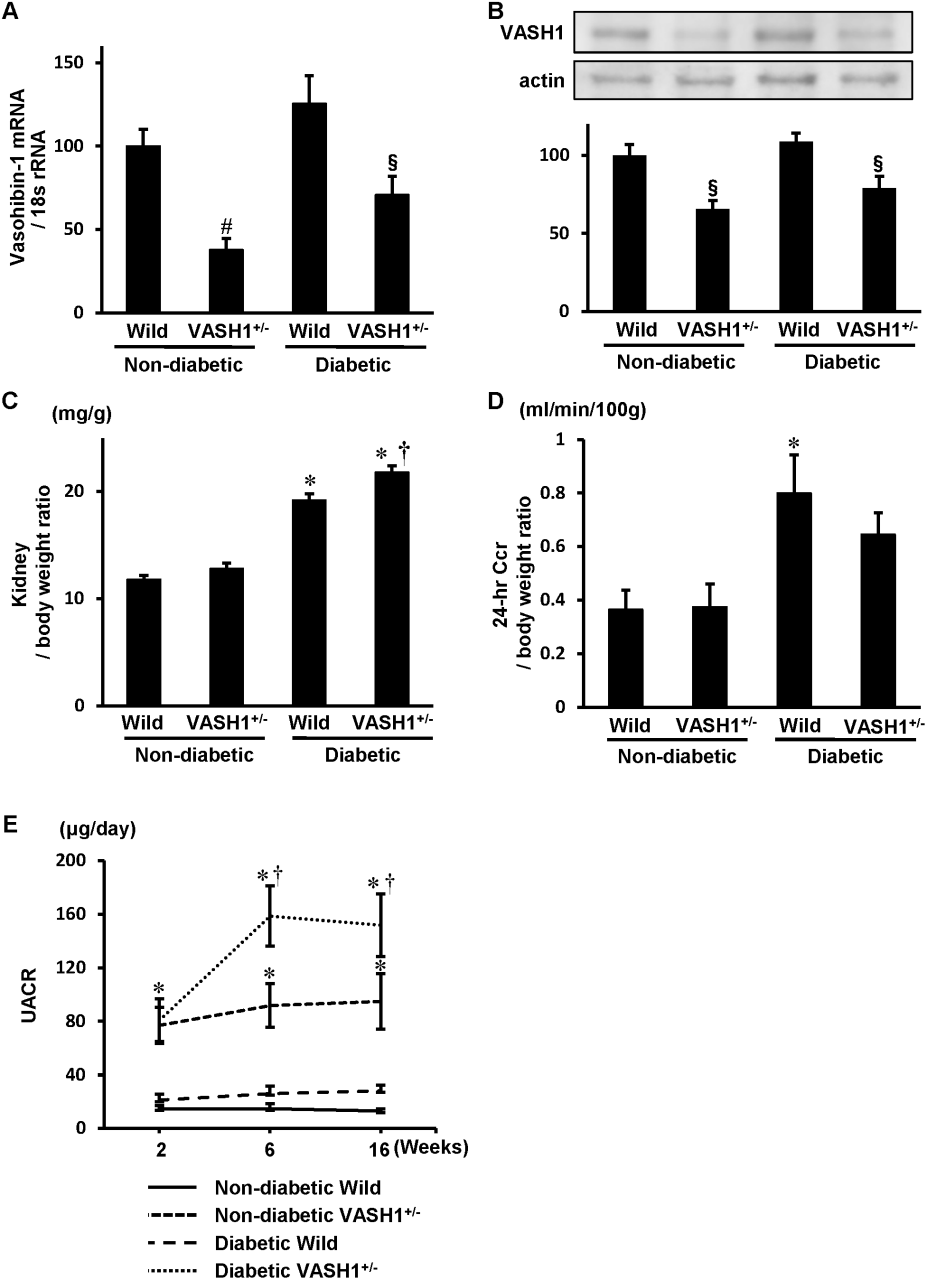
The mRNA and protein levels of Vasohibin-1, renal hypertrophy, creatinine clearance and urinary albumin excretion. *A, B*: The mRNA and protein levels of Vasohibin-1 (VASH1) detected by real-time PCR and immunoblot analysis. Total RNA and protein were extracted from the renal cortex and subjected to examinations using quantitative real-time PCR and immunoblot analysis, as described in the MATERIALS AND METHODS. Real-time PCR and immunoblot analysis showed a substantial decrease in the VASH1 mRNA and protein expression in the kidneys in the VASH1^+/−^ mice compared with the wild-type mice. The amount of VASH1 mRNA relative to 18s rRNA is shown in A. The amount of VASH1 protein relative to actin is shown in B. *n* = 4 for each group. The results of real-time PCR and immunoblot analysis are expressed relative to non-diabetic wild-type mice that were arbitrarily assigned a value of 100. *C*: The increase in the kidney weight-to-body weight ratio induced by high glucose was exacerbated in the VASH1^+/−^ mice. The kidney weight relative to the body weight was determined before termination of the experiments. *D*: The increase in the Ccr level induced by high glucose was partially reduced in the VASH1^+/−^ mice. *E*: At two, six and 16 weeks after STZ injection, the albuminuria of the diabetic mice was significantly exacerbated compared with that in the non-diabetic mice. At six and 16 weeks after STZ injection, the albuminuria of the diabetic VASH1^+/−^ mice was significantly exacerbated compared to that in the diabetic wild-type mice. n = 6 for non-diabetic Wild, 5 for non-diabetic VASH1^+/−^, 9 for diabetic Wild, 7 for diabetic VASH1^+/−^, respectively in C, D and E. ^#^ *P*<0.01, ^§^ *P*<0.05 vs. non-diabetic or diabetic wild-type mice. *P<0.05 vs. non-diabetic wild-type or VASH1^+/−^ mice. ^†^ *P*<0.05 vs. diabetic wild-type mice. Each column shows the mean ± SE. Abbreviations: Ccr, creatinine clearance; STZ, streptozotocin; UACR, the urinary albumin/creatinine ratio; VASH1^+/−^, Vasohibin-1^+/−^ mice; Wild, wild-type mice; 24-hr, 24 hours.

The experimental protocol was approved by the Animal Ethics Review Committee of Okayama University. Male C57/BL6J mice and C57/BL6J-VASH1^+/−^ mice [27], were fed a standard pellet laboratory chow and were provided with water *ad libitum*. Type 1 diabetes was induced by low-dose STZ injection, as detailed by the NIDDK Consortium for Animal Models of Diabetic Complications-(AMDCC) protocol (available from http://www.amdcc.org), with some modification. Weight-matched eight-week-old male mice received intraperitoneal injections of STZ or citrate buffer on five consecutive days. Six days after the last injection of STZ, mice with a random blood glucose concentration over the 15.5 mmol/L were selected for experiments. 19 (11 wild-type, eight VASH1^+/−^) mice received injections of STZ and 16 (nine wild-type, seven VASH1^+/−^) mice, utilizing in the experiments as the diabetic mice, exhibited hyperglycemia in the range described above. The experimental subgroups of mice included: 1) non-diabetic wild-type, 2) non-diabetic VASH1^+/−^, 3) diabetic wild-type and 4) diabetic VASH1^+/−^ mice. In Table 1 and Figure 1C–E, we used animals with the number as follows, n = 6 for non-diabetic Wild, 5 for non-diabetic VASH1^+/−^, 9 for diabetic Wild, and 7 for diabetic VASH1^+/−^, respectively. However, we used 4 mice per each experimental group in the rest of experiments.

**Table 1:** The body weight, HbA1c, blood pressure and serum creatinine level.

Group	N	Body weight (g)	HbA1c (NGSP) (%)	SBP (mmHg)	S-Cr (mg/dL)
Wild/non-diabetic	6	29.2±0.6	3.5±0.4	103.9±3.7	0.25±0.03
VASH1^+/−^/non-diabetic	5	28.7±0.8	4.4±0.4	96.9±4.0	0.25±0.04
Wild/diabetic	9	23.9±1.0*	8.3±0.3*	109.4±3.0	0.23±0.03
VASH1^+/−^/diabetic	7	24.2±0.5*	8.2±0.4*	108.6±3.4	0.31±0.03

*P<0.05 *vs.* non-diabetic mice. The values are shown as the means ± SE. Abbreviations: NGSP, national glycohemoglobin standardization program; SBP, systolic blood pressure; S-Cr, serum creatinine; VASH1^+/−^, Vasohibin-1^+/−^ mice; Wild, wild-type mice.

### Blood and urine examination

The blood glucose level, urine samples and the body weight were evaluated every other week for 16 weeks, when a 24 hours urine sample was collected in metabolism cages. The blood glucose level was measured in tail vein blood. The serum and urinary creatinine levels and urinary albumin concentration were determined as previously described [26]. The results were expressed as the urinary albumin/creatinine ratio. The creatinine clearance (Ccr) was calculated and expressed as milliliters per minute per 100 g of body weight. The survival rate until completion of the study was 100%.

### Measurement of the blood pressure

The arterial blood pressure was measured before sacrifice using a programmable sphygmomanometer (BP-2000 Blood Pressure Analysis System for Mice and Rats; Visitech Systems Inc., Apex, NC) by the tail-cuff method as described previously [28].

### Histological analysis

At 16 weeks after the injections of STZ or buffer, the kidneys were removed, fixed in 10% buffered formalin and embedded in paraffin. Sections (4 µm) were stained with periodic acid-Schiff and Masson trichrome for light microscopic observation. Mean glomerular tuft volume was determined from the mean glomerular cross-sectional tuft area as described previously [29]. Mesangial matrix index was also determined as described previously [25], [26].

### Immunohistochemistry

Immunohistochemistry was performed using frozen sections as described previously [25], [26]. The following antibodies were used as primary antibodies: (1) polyclonal rabbit anti-type IV collagen antibody (Chemicon International, Inc., Temecula, CA); (2) polyclonal guinea pig anti-nephrin antibody (Fitzgerald, Concord, MA); (3) polyclonal rabbit anti-ZO-1 antibody (ZYMED Laboratories, Carlsbad, CA) and (4) monoclonal rat anti-CD31 antibody (Pharmingen, San Diego, CA). The glomerular accumulation of monocytes/macrophages was determined by immunohistochemistry using monoclonal rat anti-Mac-2 (lectin, galactoside-binding, soluble, 3) antibody (Cedarlane, Burlington, Ontario, Canada) as previously described [30].

Double immunofluorescent staining was performed as previously described [10], [11]. The following antibodies were used as primary antibodies: (1) polyclonal rabbit anti-phosphorylated NF-κB p65 (pNF-κB p65) antibody (Cell Signaling Technology, Danvers, MA); (2) monoclonal rat anti-CD34 antibody (Santa Cruz Biotechnology, CA).

### Transmission electron microscopy

Slit diaphragm density and the GBM thickness were studied using electron microscopy techniques as described previously [31].

### RNA extraction and quantitative real-time polymerase chain reaction (real-time PCR)

RNA extraction and real-time PCR were performed as described previously, with modifications [25], [26]. The following oligonucleotide primers specific for mouse VASH1, MCP-1, tumor-necrosis factor alpha (TNF-α), CD206, interleukin-10 (IL-10), Arginase-1 (Arg-1), nephrin and 18s rRNA were used: VASH1, 5′-ATGTGGAAGCATGTGGCCAAGATC-3′ (forward) and 5′-GTCAGTCACCAATAGCCTCATAGT-3′ (reverse); MCP-1, 5′-AAGCTGTAGTTTTTGTCACC-3′ (forward) and 5′-GGGCAGATGCAGTTTTAA-3′ (reverse); TNF-α, 5′-GTTCTATGGCCCAGACCCTCAC-3′ (forward) and 5′-GGCACCACTAGTTGGTTGTCTTTG-3′ (reverse); CD206, 5′-TCGAGACTGCTGCTGAGTCCA-3′ (forward) and 5′-AGACAGGATTGTCGTTCAACCAAAG-3′ (reverse); IL-10, 5′-GACCAGCTGGACAACATACTGCTAA-3′ (forward) and 5′-GATAAGGCTTGGCAACCCAAGTAA-3′ (reverse); Arg-1, 5′-GGGAATCTGCATGGGCAAC-3′ (forward) and 5′-GCAAGCCAATGTACACGATGTC-3′ (reverse); nephrin, 5′-TCTTCAAATGCACAGCCACCA-3′ (forward) and 5′-AAGCCAGGTTTCCACTCCAGTC-3′ (reverse); 18s rRNA, 5′-ACTCAACACGGGAAACCTCA-3′ (forward) and 5′-AACCAGACAAATCGCTCCAC-3′ (reverse).

### Immunoblot

Immunoblot assay were performed as described previously [25], [26]. The following antibodies were used as primary antibodies: polyclonal rabbit anti-VASH1 antibody [32]; polyclonal rabbit anti-TGF-β1/2/3 antibody (Santa Cruz Biotechnology); monoclonal rabbit anti-phosphorylated Smad3 (pSmad3) antibody and monoclonal rabbit anti-Smad3 antibody (Cell Signaling Technology); polyclonal rabbit anti-VEGF-A antibody (Santa Cruz Biotechnology); polyclonal rabbit anti-Angiopoietin-1 (Ang-1) antibody and polyclonal rabbit anti-Angiopoietin-2 (Ang-2) antibody (Alpha Diagnostic, San Antonio, TX); polyclonal rabbit anti-IκBα antibody (Santa Cruz Biotechnology); monoclonal rabbit anti-phosphorylated IκBα (pIκBα) antibody (Cell Signaling Technology); polyclonal rabbit anti-ZO-1 antibody (Invitrogen) and polyclonal rabbit anti-beta actin antibody (Abcam).

### Cell culture

Conditionally immortalized mouse podocytes, a generous gift from Prof. Peter Mundel, were utilized to determine the direct influence of endogenous VASH1 on the high glucose-induced alterations of the mRNA level of nephrin and the protein levels of VEGF-A, Ang-1 and ZO-1, which the primary antibodies are same as above, as described previously [20], [26]. VASH1 siRNA or control siRNA were utilized to knock down endogenous VASH1. The nucleotide sequences of VASH1 or control siRNAs used in this study are as follow: for mouse VASH1 and its control, 5′-UGG UAU GGG AAU CUU GGG CAG GUC G-3′ and 5′-CGA CCU GCC CAA GAU UCC CAU ACC A-3′, respectively.

### Statistical analyses

All values are expressed as the means +/− standard error (SE). A Kruskal-Wallis test with *post-hoc* comparisons using Scheffe’s test was employed for inter-group comparisons of multiple variables. The statistical analysis was performed using the JMP version 9 software program (SAS Institute Inc, Cary, NC, USA). A level of *P*<0.05 was considered to be statistically significant.

## Results

### Exacerbated renal hypertrophy and urinary albumin excretion in the diabetic VASH1^+/−^ mice

Real-time PCR and immunoblot analysis showed a substantial decrease in the VASH1 mRNA/protein levels in the renal cortex of the VASH1^+/−^ mice compared to their wild-type littermates (**Figure 1** **, panel A and B**). The body weight (BW) was significantly lower and the HbA1c was significantly higher in all of the diabetic groups compared with the non-diabetic groups, and the diabetic VASH1^+/−^ mice did not show any significant differences in the BW, HbA1c, systolic blood pressure or serum creatinine compared with the diabetic wild-type mice **(** **Table 1** **)**. The diabetic wild-type mice exhibited marked renal hypertrophy, and this was significantly enhanced in the diabetic VASH1^+/−^ mice (**Figure 1** **, panel C**). The diabetic wild-type mice, but not the diabetic VASH1^+/−^ mice, exhibited a significantly increased Ccr/BW compared to the non-diabetic mice (**Figure 1** **, panel D**). At six and 16 weeks after STZ injection, the albuminuria of the diabetic VASH1^+/−^ mice was significantly exacerbated compared with that in the diabetic wild-type mice (**Figure 1** **, panel E**).

### Exacerbated glomerular alteration in the diabetic VASH1^+/−^ mice

**Figure 2:**
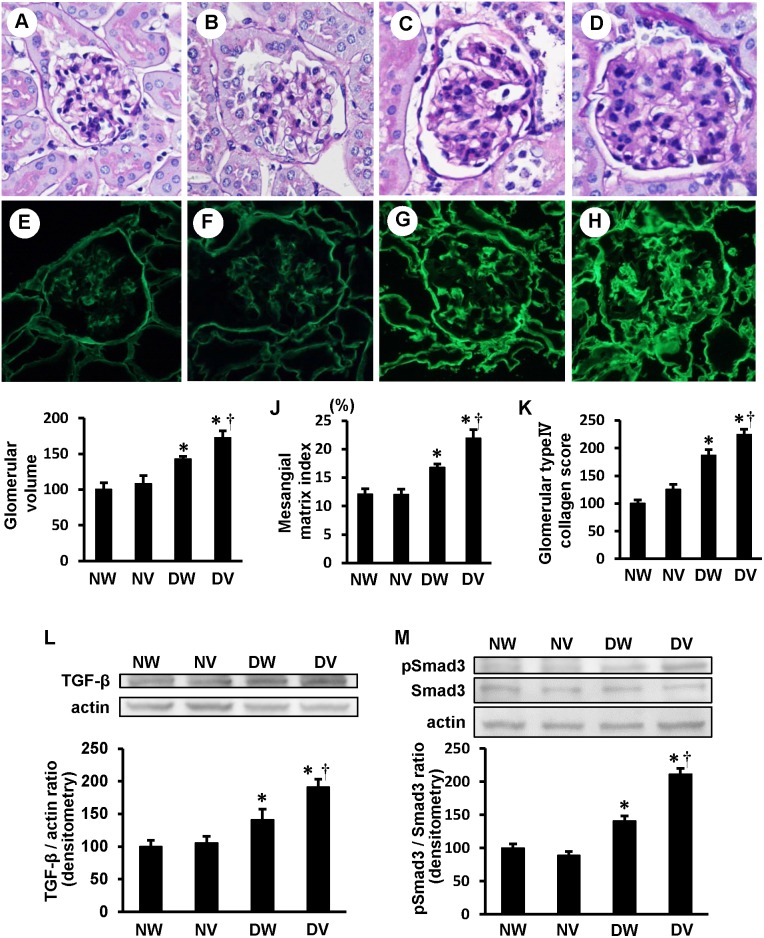
Enhanced accumulation of mesangial matrix and renal TGF-β/pSmad3 in the diabetic VASH1^+/−^ mice. *A–D*: Representative light microscopic images of glomeruli (periodic acid-Schiff staining, original magnification x400) from non-diabetic wild-type (*A*), non-diabetic VASH1^+/−^ (*B*), diabetic wild-type (*C*) and diabetic VASH1^+/−^ (*D*) mice. *E–H*: The glomerular accumulation of type IV collagen was assessed by the indirect immunofluorescence method for non-diabetic wild-type (*E*), non-diabetic VASH1^+/−^ (*F*), diabetic wild-type (*G*) and diabetic VASH1^+/−^ (*H*) mice. *E–H:* Original magnification x400. *I–K*: The increases in the glomerular volume, mesangial matrix index and type IV collagen induced by high glucose were exacerbated in the VASH1^+/−^ mice. The mesangial matrix index was defined as the proportion of the glomerular tuft occupied by the mesangial matrix area (excluding nuclei). The amount of immunoreactive type IV collagen in the glomeruli relative to the non-diabetic wild-type mice is shown (*K*). *L and M*: Immunoblots for TGF-β, phosphorylated Smad3 (pSmad3), Smad3 and actin are shown. *L (lower panel)*: The intensity of the TGF-β protein relative to actin is shown. *M (lower panel)*: The intensity of pSmad3 relative to Smad3 is shown. Each lane was loaded with 50 µg of protein obtained from the renal cortex. Each band was scanned and subjected to a densitometric analysis. *P<0.05 vs. non-diabetic wild-type or VASH1^+/−^ mice. ^†^ *P*<0.05 vs. diabetic wild-type mice. The results of glomerular volume, type IV collagen score and immunoblots are expressed relative to non-diabetic wild-type mice that were arbitrarily assigned a value of 100. Each column shows the mean ± SE. *n* = 4 for each group. Abbreviations: DV, diabetic Vasohibin-1^+/−^ mice; DW, diabetic wild-type mice; NV, non-diabetic Vasohibin-1^+/−^ mice; NW, non-diabetic wild-type mice.

Glomerular hypertrophy and an increase in the mesangial matrix index were significantly exacerbated in the diabetic VASH1^+/−^ mice compared with the diabetic wild-type mice (**Figure 2** **, panels A–D, I and J**). Focal interstitial fibrosis accompanied by tubular atrophy and thickened vessel walls was observed in the diabetic groups (Masson trichrome). No significant differences were observed between the diabetic wild-type and the diabetic VASH1^+/−^ mice (data not shown). The glomerular accumulation of type IV collagen (**Figure 2** **, panels E–H**) was significantly exacerbated in the diabetic VASH1^+/−^ mice (**Figure 2** **, panel H and K**) compared with the diabetic wild-type mice (**Figure 2** **, panel G**). Immunoreactivity of type IV collagen in the diabetic mice was observed mainly in the glomerular basement membrane and mesangial area. The diabetic mice exhibited increased renal levels of TGF-β and pSmad3 compared with the non-diabetic mice (as determined by immunoblots). The increase of renal TGF-β and pSmad3 was significantly exacerbated in the diabetic VASH1^+/−^ mice compared with the diabetic wild-type mice (**Figure 2** **, panels L and M**).

### Podocyte injuries were exacerbated in the VASH1^+/−^ mice

**Figure 3:**
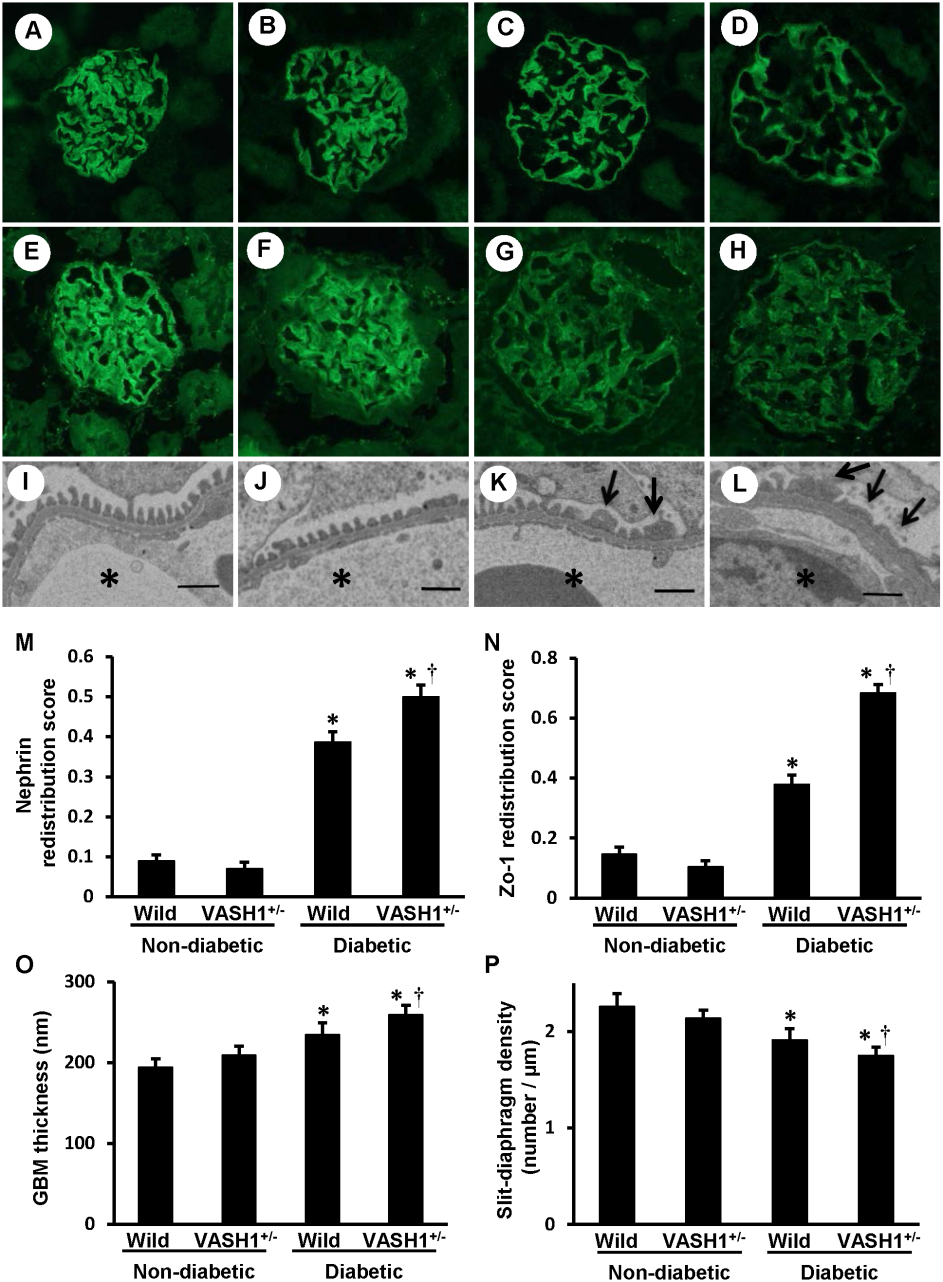
Accelerated podocyte injuries in the diabetic VASH1^+/−^ mice. *A–D*: Immunofluorescent staining of nephrin. The distribution of nephrin was determined by an indirect immunofluorescence technique in non-diabetic wild-type (*A*), non-diabetic VASH1^+/−^ (*B*), diabetic wild-type (*C*) and diabetic VASH1^+/−^ (*D*) mice. Original magnification x400. *E–H*: Immunofluorescent staining of ZO-1. The distribution of ZO-1 was determined by an indirect immunofluorescence technique in non-diabetic wild-type (*E*), non-diabetic VASH1^+/−^ (*F*), diabetic wild-type (*G*) and diabetic VASH1^+/−^ (*H*) mice. Original magnification x400. *I–L*: TEM showed the ultrastructural features, including GBM thickening, foot process effacement and fusion in non-diabetic wild-type (*I*), non-diabetic VASH1^+/−^ (*J*), diabetic wild-type (*K*) and diabetic VASH1^+/−^ (L) mice. Asterisks, capillary lumen; arrows, foot process fusion. Scale bars, 1 µm. *M and N*: The staining scores for nephrin and ZO-1 are shown as “redistribution scores”. The staining patterns of nephrin and ZO-1 were evaluated using the method described in the MATERIALS AND METHODS. *O and P:* The TEM morphometry of the GBM thickness and slit-diaphragm density. *P<0.05 vs. non-diabetic wild-type or non-diabetic VASH1^+/−^ mice. ^†^ *P*<0.05 vs. diabetic wild-type mice. Each column shows the mean ± SE. *n* = 4 for each group. Abbreviations: GBM, glomerular basement membrane; TEM, transmission electron microscopy; VASH1^+/−^, Vasohbin-1^+/−^ mice; Wild, wild-type mice.

In the non-diabetic mice, the localization of nephrin (**Figure 3** **, panels A and B**) and ZO-1 (**Figure 3** **, panels E and F**), slit diaphragm related proteins, were observed along the glomerular capillary wall in a continuous pattern. In the diabetic mice, the intensity of nephrin and ZO-1 immunostaining was diminished, exhibiting a discontinuous pattern, and thus suggesting podocyte injury (**Figure 3** **, panels C, D, G and H**). In the diabetic VASH1^+/−^ mice (**Figure 3** **, panels D and H**), the intensity of nephrin and ZO-1 was diminished, and exhibited a more discontinuous pattern compared with the diabetic wild-type mice (**Figure 3** **, panels C and G**) as confirmed by a quantitative morphometric analysis (**Figure 3** **, panels M and N**).

In addition, a significant increase in the GBM thickness and a decrease in the slit diaphragm density in the diabetic mice (**Figure 3** **, panels I–L**) were observed by electron microscopy. These alterations were exacerbated in the diabetic VASH1^+/−^ mice (**Figure 3** **, panel L**) compared with the diabetic wild-type mice (**Figure 3** **, panel K**), as confirmed by a quantitative morphometric analysis (**Figure 3** **, panels O and P**).

### Accelerated glomerular endothelial alterations in the diabetic VASH1^+/−^ mice

**Figure 4:**
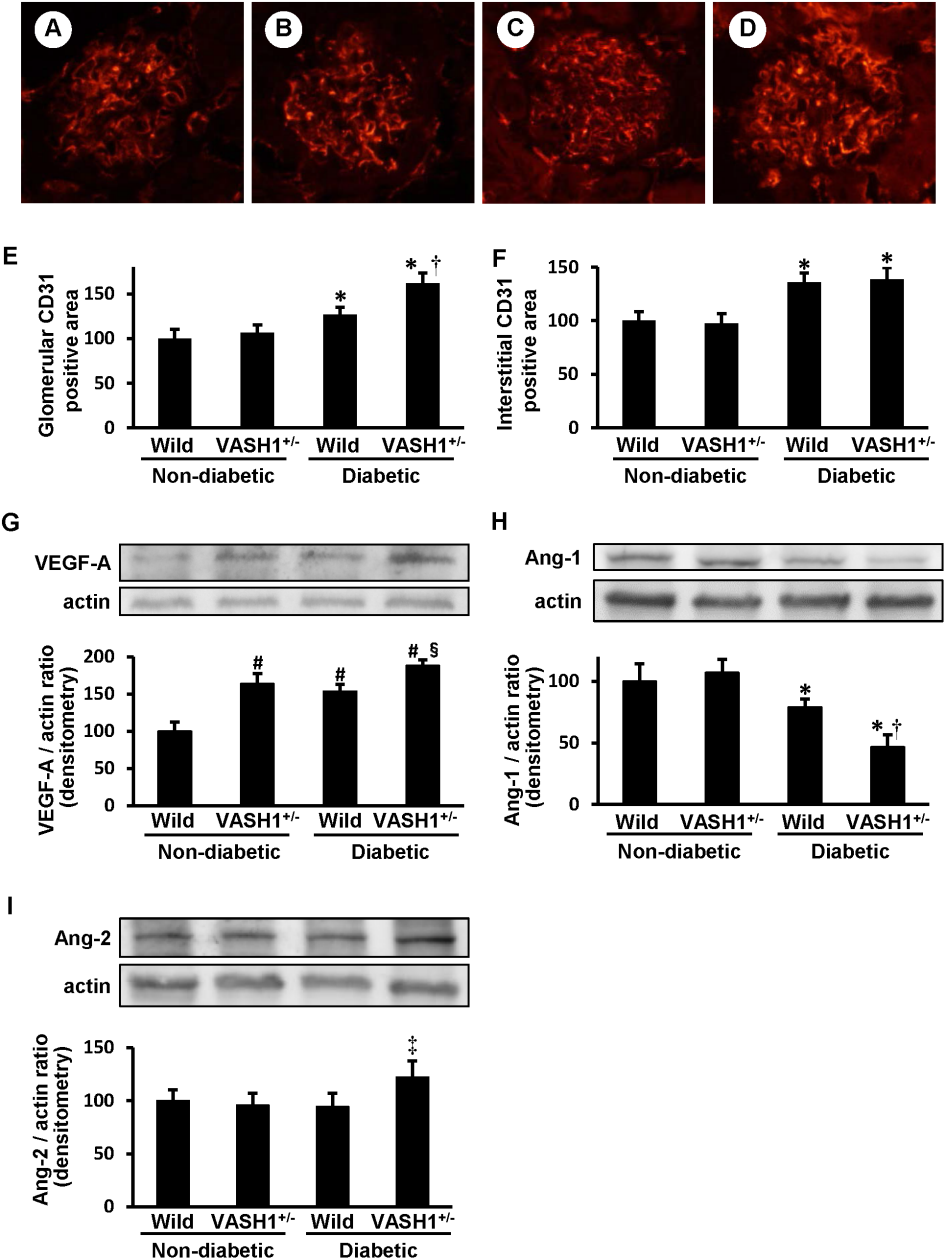
The alterations of endothelial cells and angiogenic factors in the diabetic VASH1^+/−^ mice. *A–D*: The distribution of CD31, a marker for endothelial cells, was determined by an indirect immunofluorescence technique in non-diabetic wild-type (*A*), non-diabetic VASH1^+/−^ (*B*), diabetic wild-type (*C*) and diabetic VASH1^+/−^ (*D*) mice. Original magnification x400. *E*: The glomerular CD31^+^ endothelial area was quantitated. *F*: The CD31^+^ peritubular capillary density was quantitated. *G–I*: Immunoblots for VEGF-A, angiopoietin (Ang)-1, Ang-2 and actin are shown. Each lane was loaded with 50 µg of protein obtained from the renal cortex. Each band was scanned and subjected to a densitometric analysis. *G (lower panels)*: The intensity of the VEGF-A protein relative to actin is shown. *H (lower panels)*: The intensity of Ang-1 relative to actin is shown. *I (lower panels)*: The intensity of Ang-2 relative to actin is shown. *P<0.05 vs. non-diabetic wild-type or VASH1^+/−^ mice. ^†^ *P*<0.05 vs. diabetic wild-type mice. ^#^ *P*<0.05 vs. non-diabetic wild-type mice. ^§^ *P*<0.05 vs. non-diabetic VASH1^+/−^ or diabetic wild-type mice. ^‡^ *P*<0.05 vs. non-diabetic wild-type, non-diabetic VASH1^+/−^ or diabetic wild-type mice. The results are expressed relative to non-diabetic wild-type mice that were arbitrarily assigned a value of 100. Each column shows the mean ± SE. *n* = 4 for each group. Abbreviations: VASH1^+/−^, Vasohibin-1^+/−^ mice; Wild, wild-type mice.

In the non-diabetic mice, CD31, a marker for endothelial cells, was detected along the glomerular capillaries (**Figure 4** **, panels A and B**), and was increased in the glomeruli of the diabetic mice (**Figure 4** **, panels C and D**). The glomerular CD31^+^ area was significantly increased in the diabetic VASH1^+/−^ mice (**Figure 4** **, panels D and E**) compared with the diabetic wild-type mice (**Figure 4** **, panel C**). Although the peritubular capillary (PTC) density was increased in the diabetic mice compared with the non-diabetic mice, no significant difference was observed between the diabetic wild-type and VASH1^+/−^ mice (**Figure 4** **, panel F**).

VEGF-A not only promotes vessel growth, but also promotes inflammation. Ang-1 maintains the vascular integrity through promoting pericyte attachment, but Ang-2 promotes endothelial cell activation [33]. The renal level of VEGF-A was significantly increased in the non-diabetic VASH1^+/−^ mice and the diabetic wild-type mice compared with the non-diabetic wild-type mice, and was further elevated in the diabetic VASH1^+/−^ mice as detected by immunoblot assays (**Figure 4** **, panel G**). The renal level of Ang-1 was significantly decreased in the diabetic wild-type mice compared with the non-diabetic mice, and was further diminished in the diabetic VASH1^+/−^ mice (**Figure 4** **, panel H**). The level of Ang-2 was significantly elevated in the diabetic VASH1^+/−^ mice compared with the other experimental groups (**Figure 4** **, panel I**).

### Renal inflammation was exacerbated in the diabetic VASH1^+/−^ mice

**Figure 5:**
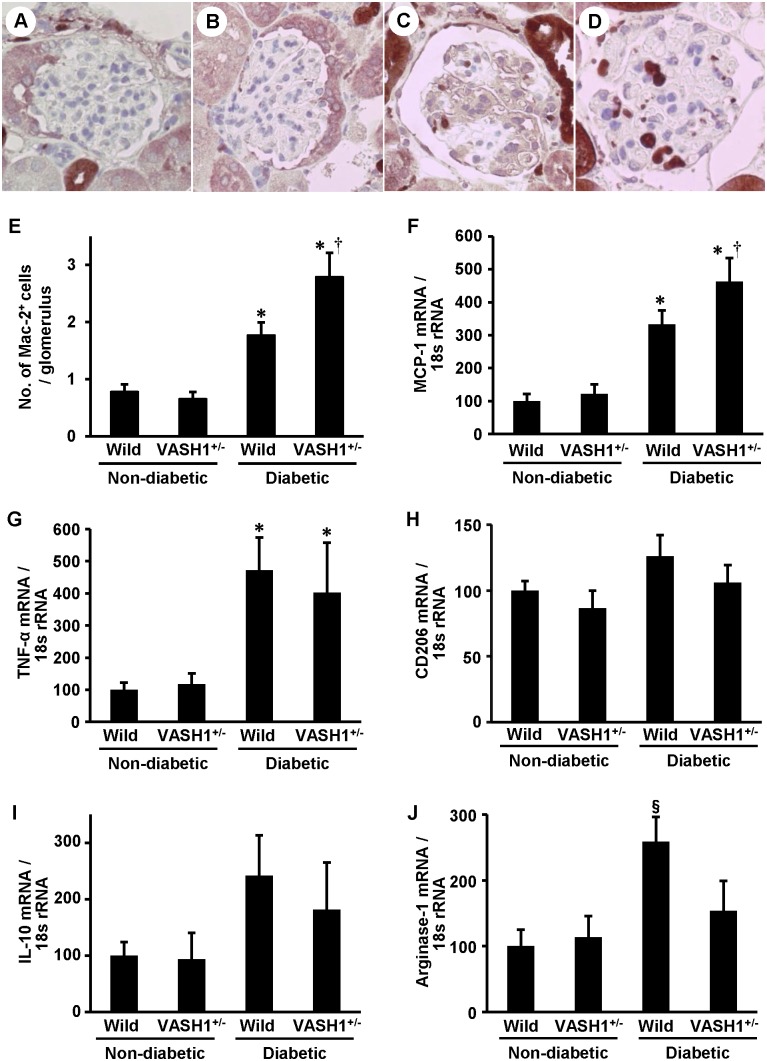
Enhanced glomerular monocyte/macrophage infiltration in the diabetic VASH1^+/−^ mice. *A–D*: The results of the immunohistochemical analysis of Mac-2^+^ monocytes/macrophages. The representative light microscopic appearance of the glomeruli in non-diabetic wild-type (*A*), non-diabetic VASH1^+/−^ (*B*), diabetic wild-type (*C*) and diabetic VASH1^+/−^ (*D*) mice are shown. Original magnification x400. *E*: The number of glomerular Mac-2^+^ monocytes/macrophages is shown. *F–J*: The mRNA levels of MCP-1 (*F*), TNF-α (*G*), CD206 (*H*), IL-10 (*I*) and arginase-1 (*J*) were detected by real-time PCR (renal cortex). The amount of each mRNA relative to 18s rRNA is shown. *P<0.05 vs. non-diabetic wild-type or VASH1^+/−^ mice. ^†^ *P*<0.05 vs. diabetic wild-type mice. ^§^ *P*<0.05 vs. non-diabetic wild-type, non-diabetic VASH1^+/−^ or diabetic VASH1^+/−^ mice. The results of real-time PCR are expressed relative to the non-diabetic wild-type mice arbitrarily assigned a value of 100. Each column shows the mean ± SE. *n* = 4 for each group. Abbreviations: No., number; VASH1^+/−^, Vasohibin-1^+/−^ mice; Wild, wild-type mice.

**Figure 6:**
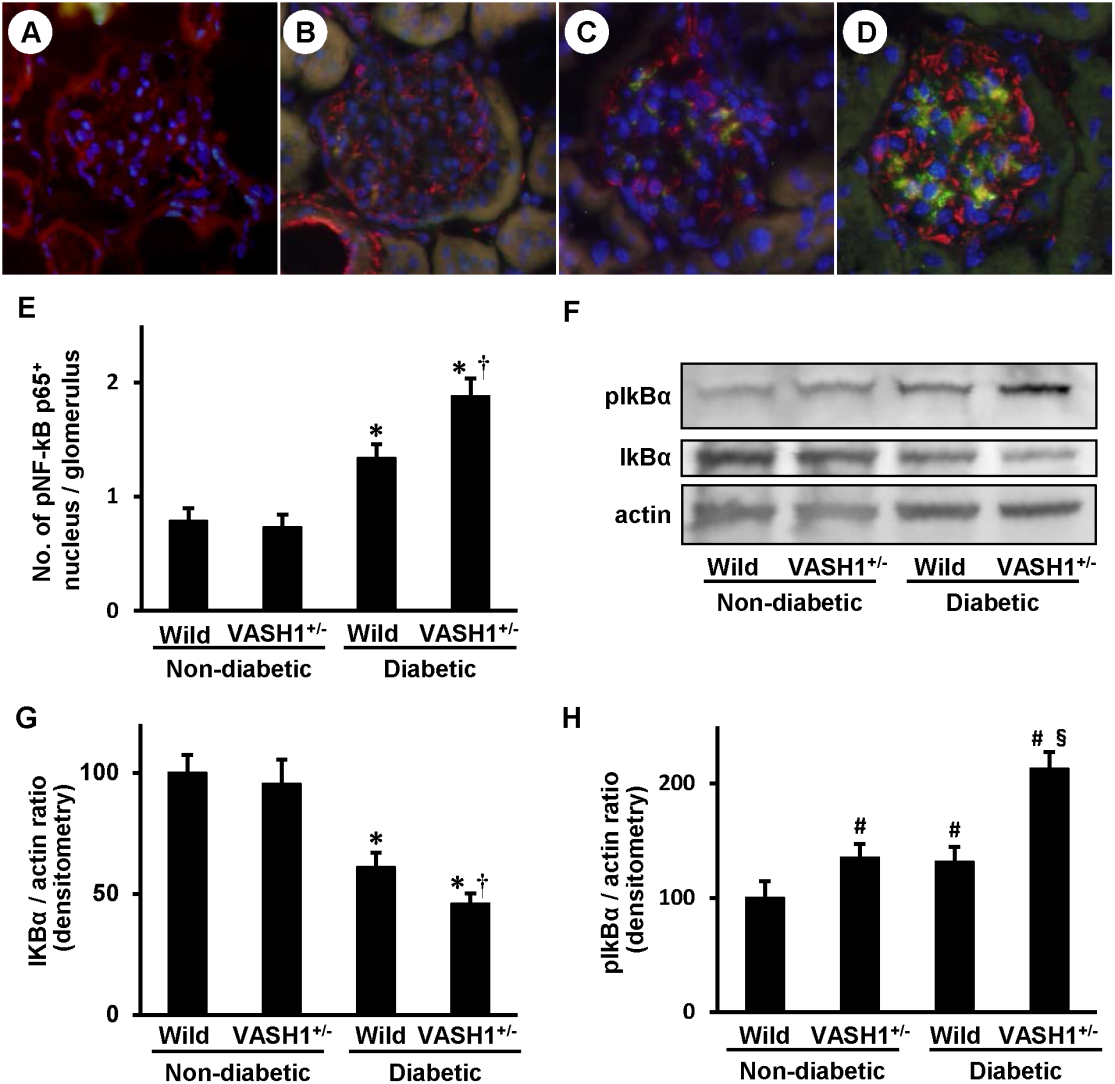
Enhanced activation of NF-κB pathway in the diabetic VASH1^+/−^ mice. *A–D:* Double immunofluorescent staining of phosphorylated NF-κB p65^+^ (pNF-κB p65^+^) (green) and CD34 (red), a marker for endothelial cells, and merged images of the glomeruli from non-diabetic wild-type (*A*), non-diabetic VASH1^+/−^ (*B*), diabetic wild-type (*C*) and diabetic VASH1^+/−^ (*D*) mice. Original magnification x400. Although pNF-κB p65^+^ was faintly observed in non-diabetic glomeruli, increased immunoreactivity for pNF-κB p65^+^ was observed, and it was co-localized with the DAPI^+^ (blue) nucleus in the diabetic wild-type mice, and this was further increased in the diabetic VASH1^+/−^ mice. *E*: The number of glomerular pNF-κB p65^+^ nuclei is shown. *F*: Immunoblots for phosphorylated IκBα (pIκBα), IκBα and actin are shown. Each lane was loaded with 50 µg of protein obtained from the renal cortex. Each band was scanned and subjected to a densitometric analysis. *G*: The intensity of the IκBα protein relative to actin is shown. *H:* The intensity of the pIκBα protein relative to actin is shown. *P<0.05 vs. non-diabetic wild-type or VASH1^+/−^ mice. ^†^ *P*<0.05 vs. diabetic wild-type mice. ^#^ *P*<0.05 vs. non-diabetic wild-type mice. ^§^ *P*<0.05 vs. non-diabetic VASH1^+/−^ or diabetic wild-type mice. The results of immunoblots are expressed relative to the non-diabetic wild-type mice arbitrarily assigned a value of 100. Each column shows the mean ± SE. *n* = 4 for each group. Abbreviations: No., number; VASH1^+/−^, Vasohibin-1^+/−^ mice; Wild, wild-type mice.

We next examined the glomerular infiltration of monocytes/macrophages utilizing immunohistochemistry of Mac-2. The number of glomerular Mac-2^+^ cells was significantly increased in the diabetic wild-type mice (**Figure 5** **, panels C and D**), compared with the non-diabetic mice (**Figure 5** **, panels A and B**), and was further increased in the diabetic VASH1^+/−^ mice (**Figure 5** **, panels D and E**). Next, the influence of VASH1 deficiency on the renal levels of M1 or M2 macrophage-associated factors was examined by real-time PCR. In the diabetic mice, the mRNA levels of MCP-1 and TNF-α, M1 cytokines, were significantly increased compared with those in the non-diabetic mice (**Figure 5** **, panels F and G**). In the diabetic VASH1^+/−^ mice, the mRNA level of MCP-1 was significantly elevated compared with that in the diabetic wild-type mice (**Figure 5** **, panel F**). The mRNA levels of CD206 and IL-10, an M2 marker and cytokine, respectively, did not significantly differ among the experimental groups (**Figure 5** **, panels H and I**). The mRNA level of arginase-1, an M2 cytokine, was significantly increased in the diabetic wild-type mice, compared with the non-diabetic mice, and was suppressed in the diabetic VASH1^+/−^ mice (**Figure 5** **, panel J**). Members of the nuclear factor κB (NF-κB) family of transcription factors are involved in inflammation and apoptosis. In resting cells, NF-κB, a heterodimer consisting of p50 and p65 subunits, is inactive in the cytosol because it is associated with nuclear factor of kappa light polypeptide gene enhancer in B cells alpha (IκBα), an inhibitor of NF-κB. At the time of cellular activation, the beta subunit of the IκB kinase complex (IKKβ) phosphorylates the inhibitor IκBα, which thereby becomes degraded and liberates NF-κB for translocation into the nucleus, where it can activate the transcription of inflammatory genes [34]. In the diabetic wild-type mice (**Figure 6** **, panel C**), the number of glomerular cells positive for pNF-κB p65 (green) in the nuclei was significantly increased compared with the non-diabetic mice (**Figure 6** **, panels A and B**), and was further increased in the diabetic VASH1^+/−^ mice, mainly in glomerular endothelial cells (CD34: red) and presumably in the mesangial cells as well (**Figure 6** **, panels D and E**). The level of IκBα was significantly decreased in the diabetic mice compared with the non-diabetic mice, and was further decreased in the diabetic VASH1^+/−^ mice, as detected by an immunoblot analysis (**Figure 6** **, panels F and G**). The level of pIκBα was significantly elevated in the non-diabetic VASH1^+/−^ mice and the diabetic wild-type mice compared with the non-diabetic wild-type mice, and further increased in the diabetic VASH1^+/−^ mice (**Figure 6** **, panels F and H**).

### The influence of VASH1 knockdown in cultured mouse podocytes

**Figure 7:**
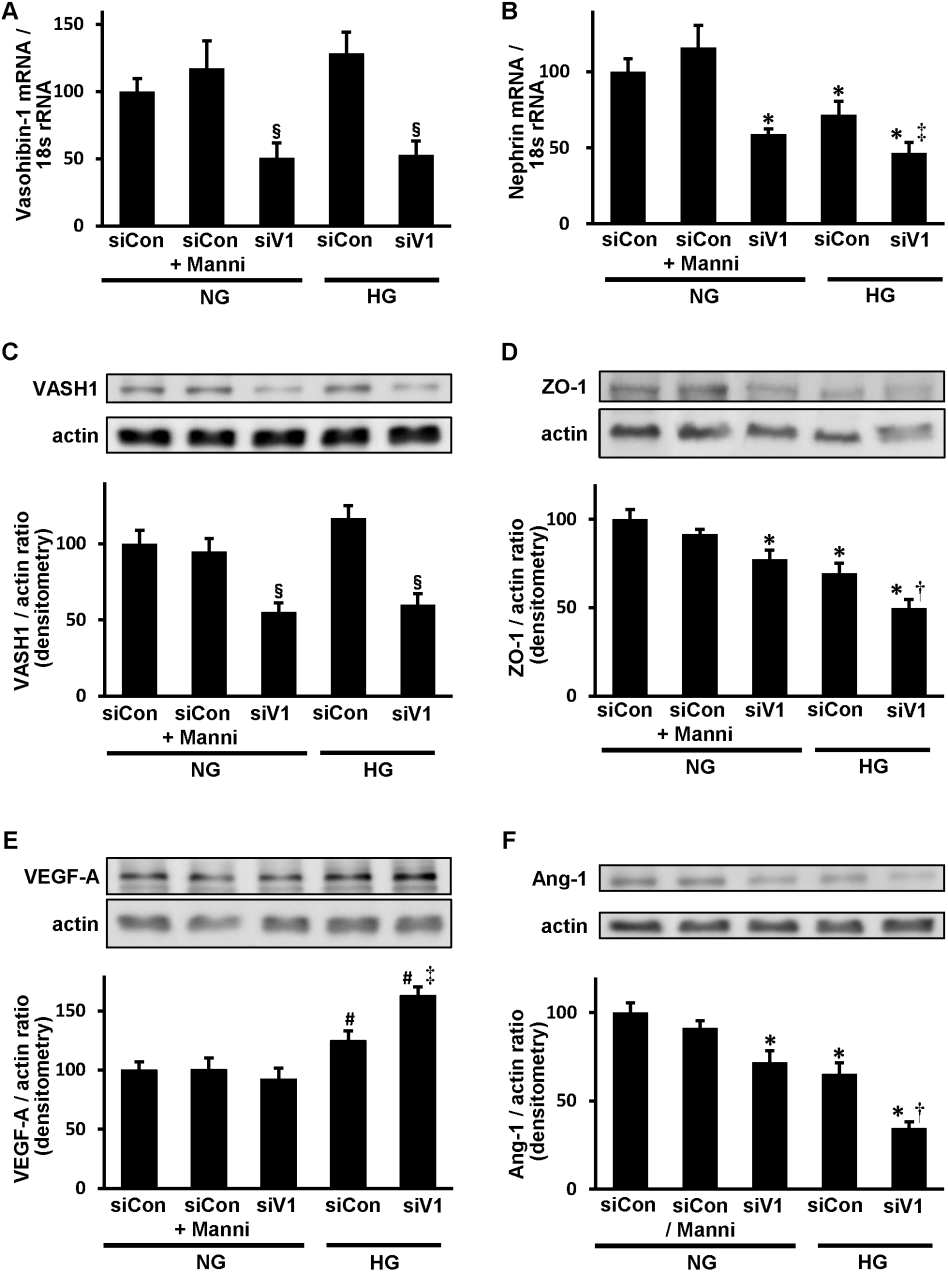
The influence of Vasohibin-1 knockdown on slit proteins and angiogenesis-related factors in cultured podocyte. Cells were cultured under normal glucose (NG; 5.5 mM), NG+Mannitol (normal D-glucose plus D-mannitol; 19.5 mM) or high glucose (HG; 25 mM) condition for 24 hours in the presence of control siRNA (siCon; 10 nM) or VASH1 siRNA (siV1; 10 nM). *A and B*: The amounts of Vasohibin-1 (VASH1) (*A*) and nephrin (*B*) mRNA relative to 18S rRNA are shown. *C–F*: Immunoblots for VASH1, ZO-1, VEGF-A, angiopoietin-1 (Ang-1) and actin are shown. In each lane, 20 µg of protein obtained from cultured mouse podocytes was loaded. The intensities of VASH1 (*C*), ZO-1 (*D*), VEGF-A (*E*) and Ang-1 (*F*) protein relative to actin are shown. ^§^ *P*<0.05 vs. control siRNA (NG, NG+Mannitol (Manni) or HG). *P<0.05 vs. control siRNA (NG or NG+Manni). ^‡^ *P*<0.05 vs. control siRNA (HG). ^†^P<0.05 vs. VASH1 siRNA (NG) or control siRNA (HG). ^#^ *P*<0.05 vs. control siRNA (NG or NG+Manni) or VASH1 siRNA (NG). The results were expressed relative to the cells cultured with NG and control siRNA that were arbitrarily assigned a value of 100. Each column shows the mean ± SE. *n* = 4 for each group.

We next performed a cell culture analysis using mouse podocytes to examine the influence of VASH1 knockdown on the podocyte integrity. After 24 hours under normal glucose (NG) or high glucose (HG) condition, transfection with the VASH1 small interfering RNA (siRNA) decreased the levels of endogenous VASH1 mRNA and protein by 50% compared with the nonspecific negative control siRNA (control siRNA) under NG condition (**Figure 7** **, panel A and C**). Since the expression of nephrin is hardly detectable in cultured mouse podocytes, we induced the expression of nephrin by culturing cells with 1, 25(OH)_2_D_3_ and all-trans-retinoic acid, as described previously [35]. When the cells were incubated with either the VASH1 siRNA under NG condition or the control siRNA under HG condition, the nephrin mRNA levels were significantly reduced compared with the cells treated with the control siRNA under NG condition. Under HG condition, treatment with the VASH1 siRNA resulted in a significant reduction of the nephrin mRNA levels compared with those observed in cells cultured with the control siRNA (**Figure 7** **, panel B**). Similar results were observed for the protein levels of ZO-1, as detected by an immunoblot analysis (**Figure 7** **, panel D**).

The HG condition significantly increased the VEGF-A levels compared with the NG condition, and treatment with the VASH1 siRNA resulted in a further increase in the VEGF-A levels compared with the control siRNA (**Figure 7** **, panel E**). Treatment with either the VASH1 siRNA under NG condition or the control siRNA under HG condition led to a significant reduction of the levels of Ang-1 compared with the group receiving the control siRNA under NG condition. Under HG condition, treatment with the VASH1 siRNA resulted in a further reduction of Ang-1 compared with cells transfected with the control siRNA (**Figure 7** **, panel F**). The addition of mannitol to NG condition did not affect the levels of VASH1, nephrin, ZO-1, VEGF-A or Ang-1, thus excluding the potential that the effects were occurring due to an elevated osmotic pressure (**Figure 7** **, panels A–F**).

## Discussion

In the present study, we utilized a VASH1^+/−^ mouse model of streptozotocin-induced type 1 diabetes. Although renal failure is not easily reproducible in this model, some of the characteristic early alterations and histopathological changes could be observed similar to human diabetic nephropathy [36]. Although diabetic mice exhibited significant weight loss, the extent of body weight reduction was comparable to previous reports utilizing this model [25]. In the diabetic wild-type mice, albuminuria, glomerular hypertrophy, glomerular hyperfiltration (as evidenced by an increased Ccr) and renal hypertrophy were observed, consistent with previous study [37]. These abnormalities were significantly exacerbated in the diabetic VASH1^+/−^ mice compared with the diabetic wild-type mice, except for the change in the Ccr.

Podocyte injury in association with altered expression of podocyte slit proteins is involved in the development of proteinuria in diabetic nephropathy. The reduction as well as the altered localization of nephrin and ZO-1, components of the slit diaphragm cell adhesion complexes [38], in the diabetic wild-type mice were significantly exacerbated in the diabetic VASH1^+/−^ mice, partly attributable to the increased albuminuria in these mice. In addition, the augmentation of the GBM thickness and reduction of slit diaphragm density in the diabetic wild-type mice were significantly exacerbated in the diabetic VASH1^+/−^ mice. The knockdown of VASH1 by siRNA further decreased the levels of nephrin and ZO-1 under HG condition in cultured mouse podocytes. The present findings are consistent with our previous reports demonstrating the protective effects of VASH1 overexpression on the albuminuria in the diabetic db/db mice and the protective effects of recombinant human VASH1 on cultured murine podocytes under HG condition [26].

In the present study, the level of VEGF-A was increased in the renal cortex of diabetic mice and in the podocytes under HG condition, consistent with previous studies [13], [14], [17], [39], [40]. Furthermore, the renal levels of VEGF-A were significantly increased in the diabetic VASH1^+/−^ mice compared with the diabetic wild-type mice, and the VASH1 knockdown resulted in increased VEGF-A levels in cultured mouse podocytes under HG condition. The increased levels of VEGF-A in the diabetic VASH1^+/−^ mice as well as VASH1 siRNA-transfected mouse podocytes may be associated with the deterioration of diabetic nephropathy, consistent with previous reports [13], [14]. On the contrary, a recent report suggested that the upregulation of VEGF-A in diabetic kidneys might protect the microvasculature from injury [41]. Therefore, it is also possible that renal VEGF-A might be elevated to compensate for the renal injury in the diabetic VASH1^+/−^ mice.

In the normal adult glomerulus, Ang-1 is constitutively expressed in podocytes, whereas the Ang-2 level remains to be low [42]. Dysregulation of Ang-1 and Ang-2 in the glomeruli was observed in diabetic nephropathy and other glomerular diseases [43], potentially associated with endothelial injuries, hyperpermeability and albuminuria. The level of Ang-1 was significantly decreased in the diabetic wild-type mice compared with the non-diabetic mice, and was further decreased in the diabetic VASH1^+/−^ mice. The level of Ang-2 was significantly elevated in the diabetic VASH1^+/−^ mice in comparison to the other mice. Therefore, the Ang-1/Ang-2 ratio was significantly decreased in the diabetic VASH1^+/−^ mice compared with the diabetic wild-type mice, potentially associated with the inflammatory alterations [25]. VASH1 knockdown in cultured mouse podocytes under HG condition led to upregulation of VEGF-A and downregulation of Ang-1, similar to the results observed *in vivo*.

Experimental rodent diabetic models exhibit an increased glomerular filtration surface area in the early stage [10], [11]. In the diabetic VASH1^+/−^ mice, the CD31^+^ glomerular endothelial area was further increased compared with the diabetic wild-type mice, suggesting an enhanced pro-angiogenic status due to VASH1-deficiency.

The potential role of VEGF-A in mediating glomerular monocyte/macrophage infiltration has been demonstrated in diabetic animal models [44]. The exacerbation of the inflammatory alterations, namely enhanced infiltration of glomerular Mac-2^+^ cells, in the kidneys of the diabetic VASH1^+/−^ mice might be associated with the activation of VEGF-A signaling, as well as the augmentation of the renal MCP-1 levels. Consistent with this study, we previously observed the anti-inflammatory effects of exogenous VASH1 in association with the suppression of excessive VEGF-A signaling and the inhibition of the renal MCP-1 levels in experimental diabetic nephropathy [26]. Similarly, the therapeutic effects of VASH1 on the formation of the arterial neo-intima have also been reported in association with inhibitory effects on adventitial macrophage infiltration [21].

Macrophages exhibit a range of phenotypes, a phenomenon that has been described as macrophage polarization or heterogeneity [6], [45], [46]. The “classically” activated M1 macrophages, which are induced by interferon-γ, lipopolysaccharide, TNF-α or granulocyte-macrophage colony stimulating factor, express proinflammatory cytokines such as interleukin (IL)-1β, TNF-α, MCP-1 and IL-6 and play a pathogenic role in renal inflammation. In contrast, exposure of macrophages to IL-4 or IL-13 inhibits the expression of these proinflammatory cytokines, and instead activates the expression of arginase-1, mannose receptor and IL-10. These “alternatively” activated M2 macrophages modulate the inflammatory response and promote tissue repair [47], [48]. In the present study, the pro-inflammatory cytokines, such as TNF-α and MCP-1, M1 macrophage-derived, were upregulated in the diabetic wild-type mice, and the MCP-1 levels were further elevated in the diabetic VASH1^+/−^ mice. The anti-inflammatory cytokines, such as arginase-1, M2 macrophage-derived, were significantly upregulated in the diabetic wild-type mice, but not in the diabetic VASH1^+/−^ mice. Therefore, the dysregulation of the M1/M2 macrophage subpopulation may also contribute to the exacerbated renal inflammation in the diabetic VASH1^+/−^ mice. In line with these results, the phosphorylation of IκBα, which becomes degraded and liberates NF-κB for translocation into the nucleus, and the nuclear translocation of pNF-κB p65 were augmented in the kidneys of the diabetic VASH1^+/−^ mice compared with the diabetic wild-type mice, potentially associated with the exacerbated renal inflammatory alterations.

TGF-β_1_ is a key mediator of renal fibrosis [49], [50] including diabetic nephropathy [4], [5]. Smad2 and Smad3 are the critical downstream mediators responsible for the biological effects of TGF-β_1_. Furthermore, the downstream targets of TGF-β/Smad3 signaling are the collagens and tissue inhibitor of matrix metalloproteinase-1 (TIMP-1) [51]. In the diabetic VASH1^+/−^ mice, the renal levels of TGF-β and pSmad3 were significantly increased in association with the accumulation of mesangial matrix and glomerular type IV collagen. We previously reported the inhibitory effects of recombinant VASH1 on the HG-induced increase of TGF-β levels in cultured mesangial cells [25], thus suggesting the direct regulatory effects of endogenous VASH1 on mesangial cells. Podocyte-derived VEGF-A, induced by TGF-β_1_, stimulates the production of α3(IV) collagen, one of the components of the GBM [52]. Therefore, the regulatory effects of endogenous VASH1 on mesangial matrix expansion may also be mediated through the regulation of VEGF-A.

In the present study, renal levels of mouse VASH1 (mVASH1) mRNA in the diabetic wild-type mice were slightly elevated without statistical significance compared with the non-diabetic wild-type animals. These results are consistent with our previous study employing the identical type 1 diabetes model, with slight increase of renal mVASH1 levels in the control diabetic mice [25]. In our previous studies employing adenoviral vectors encoding human VASH1 (hVASH1) in the murine type 1 and type 2 diabetes models, we observed therapeutic effects on diabetic renal alterations [25], [26]. Interestingly, mVASH1 levels were not altered by adenoviral delivery of hVASH1, in contrast to hVASH1 exhibiting elevated levels in those studies. Therefore, we speculated that therapeutic effects observed in those previous studies were attributable to the exogenously administered hVASH1, rather than endogenous mVASH1.

Recently, we demonstrated that the increased plasma and urinary levels of VASH1 were significantly correlated with worse renal outcomes [53]. Similar to our findings, several previous reports had demonstrated that an elevated expression of VASH1 predicted a worse clinical outcome in patients with cancer [54], [55], [56], [57], [58], [59]. In various experimental disease models including those of cancer and diabetic nephropathy, the administration of adenoviral vectors encoding VASH1 resulted in therapeutic effects [19], [21], [22], [23], [24], [25], [26], [60]. More recent findings have demonstrated the role of VASH1 in enhancing the stress resistance of endothelial cells [20]. Therefore, we suppose that endogenous mVASH1 was upregulated in a compensatory manner in response to increased disease activities and endothelial cell stress in the present diabetic mice model. However slight elevation of endogenous mVASH1 might be insufficient to improve the diabetic renal alterations.

There are several limitations associated with the present study. First, we evaluated the regulatory role of endogenous VASH1 in a type 1 diabetic nephropathy model, and the use of distinct diabetic animal models, i.e. type 2 diabetes, should be considered in the future to verify our findings. Secondly, since we observed the functional role of endogenous VASH1 in maintaining the podocyte integrity in diabetic nephropathy, further studies utilizing podocyte-specific VASH1 knockout mice would be warranted.

In conclusion, the present results suggest that endogenous VASH1 may possess renoprotective effects against type 1 diabetic nephropathy, via regulating inflammation and fibrosis and protecting podocytes from injuries, thus indicating the potential therapeutic efficacies of VASH1 in diabetic nephropathy.
